# Supplementary material for: A Simple and Direct Assay for Monitoring Fatty Acid Synthase Activity and Product-Specificity by High-Resolution Mass Spectrometry
Source: Biomolecules. 2020 Jan 10;10(1):118. doi: 10.3390/biom10010118 (PMC7023185; doi:10.3390/biom10010118)
Supplement: Supplementary file 1 [file biomolecules-10-00118-s001.pdf]

Supplementary information

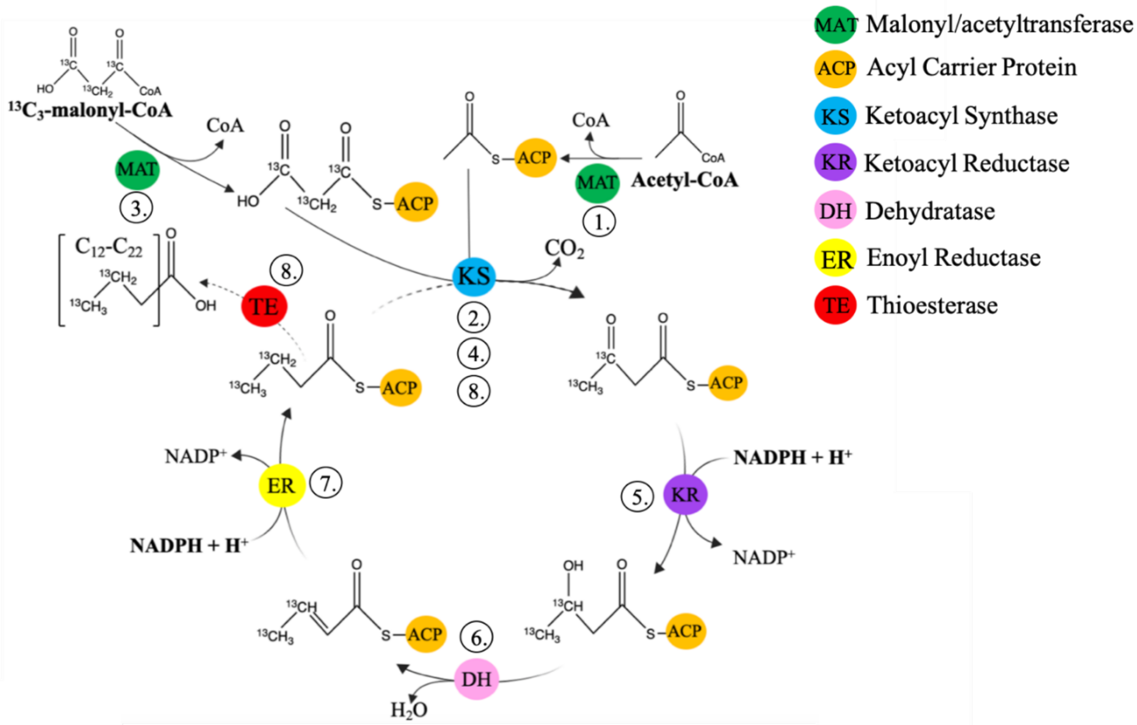

Figure S1. Overview of de novo fatty acid synthesis in mammals. Numbers denote the order of the reaction cycle: (1) Acetyl transfer onto ACP by MAT; (2) Acetyl/acyl transfer to KS; (3) malonyl transfer to ACP by MAT (exemplified with a 13C3-malonyl group); (4) condensation of acetyl/acyl group with the malonyl group by the KS; (5) ketoacyl reduction using NADPH; (6) dehydration; (7) enoyl reduction using NADPH; and (8) release of the FA chain by TE as an NEFA.

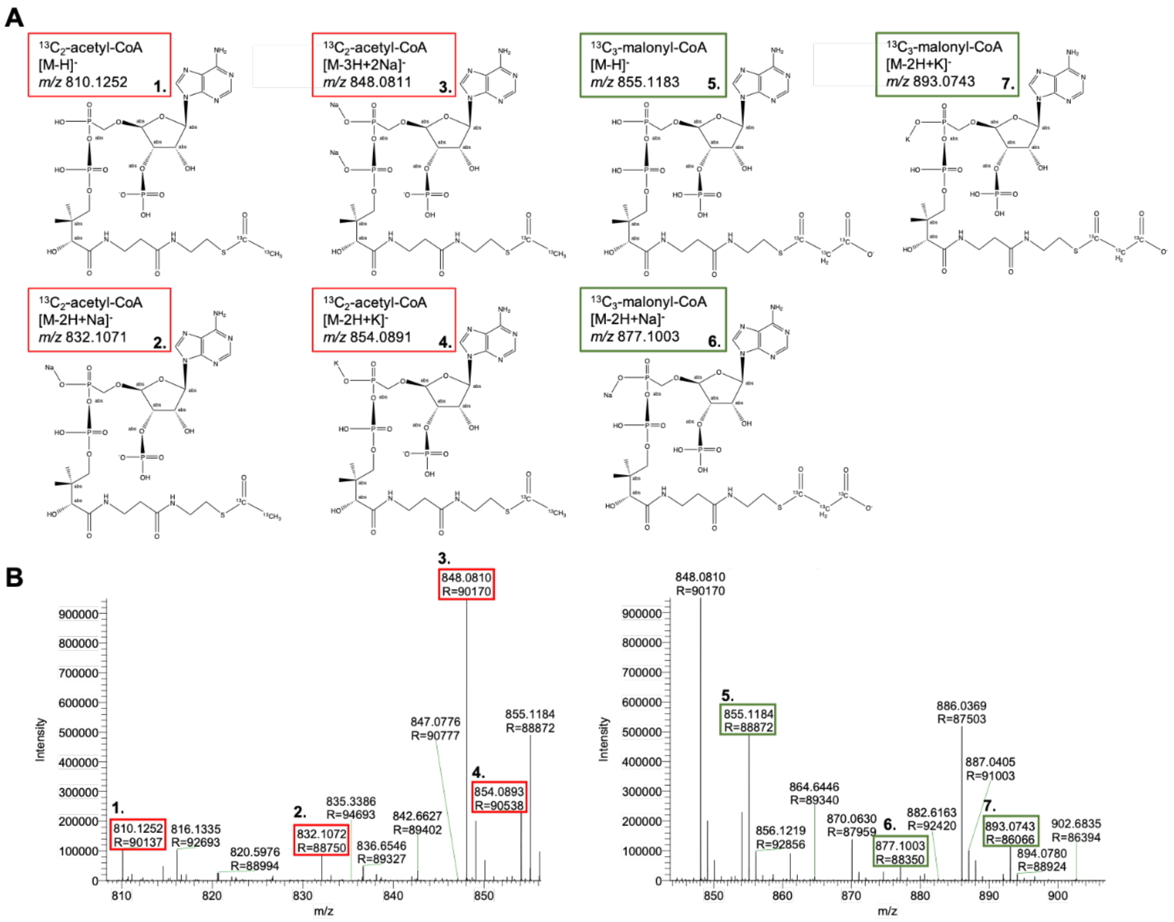

Figure S2. Negative ion mode FTMS analysis of commercially available $^{13}C_3$-malonyl-CoA. (A-B) The $^{13}C_3$-malonyl-CoA was diluted to 10 µM in methanol and directly injected using a Triversa NanoMate robotic nanoflow ion coupled to an LTQ Orbitrap XL mass spectrometer. Chemical structures of the detected $^{13}C_3$-malonyl-CoA and $^{13}C_2$-acetyl-CoA are highlighted in green and red, respectively.
